# 

**Published:** 1917-01-20

**Authors:** 


### LECTURES ON PUBLIC HEALTH.

The Royal Institute of Public Health has arranged an interesting series of lectures, to be given on Wednesdays at 4 P.M. till March 28, on "Public Health Problems under War and After-War Conditions." "Child-welfare Work," "Prevention of Venereal Diseases," "Milk Supply," and "Health of Munition Workers" are amongst the subjects chosen, and these, together with an attractive list of lecturers, including Sir Alfred Pearce-Gould, Dr. Mary Scharlieb, Lady Barrett, Professor Sir Thomas Oliver, and others, should attract a good attendance of social workers. The lecture-room of the Institute is at 37 Russell Square, W.C.

Rates of Subscription: Twelve months, 6/6; Six months, 4/-; Three months, 2/-; post free to any address in the United Kingdom. Abroad, Twelve months, 8/8; Six months, 5/-; Three months, 2/6, post free. The Hospital "Index" to Volume LX., April to September 1916, is now ready, price 2½d. per copy, post free. Address The Manager, "The Hospital," The Hospital Building, 28/29 Southampton Street, Strand, London, W.C.

